# Medical Service in the Highlands and Islands

**Published:** 1915-07-24

**Authors:** 


### MEDICAL SERVICE IN THE HIGHLANDS AND ISLANDS.

### Life and Needs of the Remote Doctors.

The life and work of the medical practitioner in the remote districts of the Highlands and Islands of Scotland have sometimes been presented to the public in a romantic or heroic guise. No one who is acquainted with the facts will deny that individual instances have afforded a substantial basis of truth for these sketches of the popular novelist. Yet it is certainly not less true that over large tracts of country medical attendance has, in the very nature of the case, been difficult to obtain, and that the conditions of medical work cannot be called attractive to the great majority of practitioners. A scattered population, considerable hindrances to easy travelling, exposure at times to severe climatic discipline, remuneration modest even to scantiness, isolation from all stimulating professional influences, and the absence of congenial society—these are not inviting conditions to ambitious and energetic youth. Now and again, by some whim or circumstance, an exceptionally capable and studious man has buried himself in an environment of this character, but obviously, and probably in an increasing measure, the inclination of the practitioner leans in quite other directions. Hence, admitting such exceptions as are here contemplated, it must be allowed that the medical service in the Highlands and Islands has long been unsatisfactory in many important respects.

### The Act of 1913.

The facts of the situation, though well known to a few, acquired some public notoriety when the National Insurance Act came into operation, and they led the Scottish Insurance Commissioners to make representations to the Treasury. As a result, a Committee was appointed, under the chairmanship of Sir John A. Dewar, Bart., M.P., to inquire into the whole matter and to advise as to the best method of securing a satisfactory medical service wherever in the Highlands and Islands the existing service was defective. In 1913 the recommendations of this Committee were embodied in an Act of Parliament, by which it was enacted that a sum of £42,000 should be provided in each financial year, and should be known as the Highlands and Islands Medical Service Fund. For the administration of the fund a special Board was created, and the first Report of this Board has just been issued. In certain respects the proposals of the Board are not yet authoritative, because they have yet to receive the approval and sanction of the Secretary for Scotland. But there are many features of the Report which are of great interest, and the time is not inappropriate to give them some attention. The people for whose assistance this new Board has been formed have many claims to consideration and sympathy. They live under conditions which only the hardy and robust may hope successfully to oppose, their picturesque country is in summer-time a holiday-ground for many whom fortune has treated with a lavish hand, and the people have generally the attraction of primitive habits and of the straightforward outlook of those who dwell apart from the busy haunts of men. At this moment, it will be recalled, from these straths and glens and stormy seas have come forth large numbers of men, not wanting in physique or in valour, to fight for King and country, and many a "pretty" man will see his Highland home no more. Few are the homes whence youth has not gone forth to fight, and there are literally whole districts where not one single man of military age can be found. The "lone shieling" and the "misty island" may well, therefore, appeal to all our sympathies. What will promote the welfare of the Highlands has a patriotic sanction, for it means the recognition of brave hearts and stout arms for the defence of our country.

To return, however, to the Report of the new Board. The task with which they were faced was not an easy one. Money, it is true, was at their disposition; but it had to be wisely used, and to secure this a supply of trustworthy information was essential. Clearly, it would have been disastrous had the arrival of the new authority conveyed to those already in existence the notion that local affairs might be neglected. Again, a mechanical distribution of the funds on the basis, say, of population or of valuation, though an easy proceeding, would have failed to recognise the special peculiarities and necessities of the individual areas. Hence, very wisely, the Board resolved to obtain first-hand and confident information. This was effected in part by a series of questions addressed to various bodies, but also and to a large extent by actual personal visits and investigations conducted on the spot. The immediate inquiries extended from March to the middle of August, and included a distinctly formidable itinerancy, which extended to many remote districts, and was helped out on its sea-borne portion by one of the cruises of the Scottish Fishery Board. Local authorities and doctors were interviewed; where possible, public meetings were arranged; and hospital and nursing arrangements, in so far as they exist, were examined on the spot. All this, it is unnecessary to say, meant very real and trying work. Hardly likely to attract much general recognition, a tribute may well be paid to it by all who are aware how easy it is to spend public money without advantage, indeed with positive harm, to the recipients. A further remark justified by these careful and minute personal investigations is that recommendations based upon them must clearly have great force and value; they arise from first-hand knowledge, and manifestly, therefore, have an authoritative quality. With regard to the actual administration of the grants to be made to the several districts the Board decided that there shall be created in each a special local authority, to consist of representatives of all the public bodies in whom Parliament has placed responsibility for dealing with matters affecting the health of the individual or of the community. A scheme for the election of these administrative Committees has, therefore, been prepared, together with suggestions relating to the principles on which the work should be conducted.

The position, stated in general terms, which had to be faced was that general medical practice in the Highlands and Islands rested very largely on the subsidy from the Poor-Law authority and to a less extent on the subsidies from other public bodies, these being in certain districts supplemented by contributions from local proprietors, though this local support in the shape of subscriptions to medical clubs and to nursing associations has recently been a diminishing quantity. The inadequacy of these arrangements was evident from the experience that in not a few districts advertisements by the parish authorities failed to secure any applications from medical practitioners; and similar difficulties attended attempts to find nurses for some of the smaller islands and outlying stations. This position in some of its aspects has been rendered more acute by the war, which has both lessened voluntary subscriptions and diminished the numbers of available doctors and nurses. Under the new proposals the Board will make a special and immediate allowance to any district where there is urgent need of medical and nursing assistance, in the hope that a larger remuneration will attract the help that is needed. One of the conditions of this special help is that the locality is willing to do what is possible in the desired direction, for the Board strongly endorses the view of the Treasury that the new grant is to be devoted to the improvement of existing conditions and not to the release of localities from the claims which they have hitherto met. Under this emergency proposal the Board has kept, or has assisted in keeping, medical service in five districts, has guaranteed a salary for a doctor in six districts, and has made a nursing service possible in eighteen districts.

### The Policy Proposed.

With regard to the policy which is to be followed in the future the following points may be noted. The local authorities will be invited to submit suggestions and requests indicating where in particular help is needed, and the Board will make grants as seems to them good, and will satisfy themselves that efficiency is secured. In particular, every effort will be made to secure available medical attendance for persons of only very limited means, and to promote such attendance without additional cost to the patient when his residence happens to be a considerable distance from the nearest doctor. Attempts will be made to encourage the possibility of communication by telegraph and telephone, and to provide the doctor with the most suitable means of conveyance—motor-car, motor-cycle, motor-boat, a horse for bridle paths, and so forth, according to circumstances. Even, if necessary, a house may be supplied, with a view to encourage a doctor to reside at a centre convenient for the population depending on his attendance. Another proposal is to establish in suitable places small hospitals capable of accommodating four to six patients and a small resident staff—say, one nurse and a maid-servant; this would help to meet the position of patients resident at a considerable distance from a doctor, and at the same time needing frequent attendances. The nurse would make the hospital her headquarters, but, when circumstances admit, she could be available for outside work, while in districts where no hospital is established provision will be made to see that efficient nursing service is at hand. It is hoped, too, that at least in some measure the more specialised branches of medical practice may be encouraged, as also opportunities for laboratory investigation. In all these respects the efforts of the Board will be to work in co-operation with local authorities, leaving to these latter detailed arrangements and the necessity of continuing their present support to the medical services already in operation.

When, however, all is done in the directions above indicated, there will remain places which are quite beyond the reach of existing organisations, and to meet these circumstances the Board intends to organise a small staff of doctors and nurses who will be immediately under their direction and control. These will have the duty of visiting at regular intervals the outlying islands and other isolated communities, and of residing there for such periods as may be deemed advisable. On such visits they will be expected not merely to treat patients who solicit their advice, but, in addition, to interest themselves in the general well-being of the community, and specially to give advice in reference to infants and school-children. Further, these official doctors would be available for relief work in the assistance of medical men in isolated districts who may happen to be laid aside, and to visit and to report on any branch of the service on which the Board desires information. At present, and in consequence of the war, the development of this proposal has been found to be impossible, though authority has been granted for the appointment of one doctor and one nurse under it. Indeed, so many of the practitioners in the areas related to the Board are already absent on military duty that special efforts have had to be made to maintain what may be called the normal medical service. It has been necessary in some districts to arrange an intermittent service in place of a regularly resident doctor, and here help has been afforded by the Scottish Medical Emergency Committee.

In dealing with the nursing service the Board will act on the same principles as those which are to govern the medical service. Existing organisations will, where necessary, be subsidised, and will be expected to reach a satisfactory level of efficiency. New centres will be encouraged, and in circumstances which call for it the Board will undertake the provision of a nurse on its own responsibility. Indeed, in some of the smaller and more remote islands the nurse will have to be the principal representative of professional assistance. A resident medical practitioner is out of the question, though, as already explained, even the most isolated community may have at regular intervals the brief stay of a doctor specially appointed by the Board.

This first Report affords a guarantee that the situation has been explored in all its aspects, and the proposals are eminently practical and to the point. For the moment, here, as elsewhere, desirable developments are hindered by the war and by the consequent shortage of medical practitioners. It may thus be some considerable time before the new proposals are in full operation. That they are calculated to improve medical attendance and care in the districts concerned is obvious, and we shall look forward with much interest to the further Reports, which will describe, we doubt not, a large success.

On the financial side the income of the fund shows a total of £84,820 18s., which includes the grant-in-aid for 1913-14 and 1914-15, interest on balance, and certain extra receipts. The expenses have been: Medical service, £625 12s. 7d.; nursing service, £475 10s. 6d., and grants to Insurance Committees towards travelling expenses of practitioners, £9,525. The balance of £74,164 14s. 11d. will, it is expected, be required to meet the capital expenditure contemplated above, and involved in the provision or improvement of an ambulance service.

